# Medicine

**Published:** 1897-03

**Authors:** Theodore Fisher, J. Michell Clarke


### Progress of the Medical Sciences.

### MEDICINE.

It is natural and justifiable that thinking members of the medical profession should look with critical eyes upon any description of a disease that is assumed to be new. Especially is that the case with an infectious disease, and many experienced practitioners will be loth to believe that still another specific fever exists, and may have passed unobserved under their notice. When, however, we read such a description, if we cannot convince ourselves that its originator has been misled by some unusual features of a previously known disease, it behoves us to bear his facts in mind, and to be on the watch for opportunities to test their value. Brief notice will be given here of the disease described during the last few years as **glandular fever**, and I trust that the readers of this *Journal* may, after noting the few points mentioned, consider whether some of their patients may not have been recently suffering from a disease which answers to the description. In 1889 an infectious disease occurring in children was described by Pfeiffer, in which the most important symptoms were swollen, tender glands in the neck, and fever.

This fever, described as glandular fever, has since been occasionally observed by continental writers, but not until the last two or three months has it been prominently brought before the notice of English-speaking people. Last December a careful description of an epidemic by Dr. Park West[1] appeared in America, and in this country Dr. Dawson Williams[2] has more recently called attention to the disease.

The paper by Dr. Park West is based on no less than ninety-six cases which occurred in the practice of Dr. Korell of Ohio. Dr. West thus describes some of the leading characteristics: "The most marked feature in all the cases was the enlargement of the cervical, or to be more definite, the carotid, lymphatic glands. After two to three days of *malaise* the swelling could be seen. As a rule it began on the left side and reached full development on the second to fourth day; several hours before its completion on this side it could be noticed on the right, and the same course would be followed as on the left. Occasionally the swelling began on the right side, but in no case did it appear simultaneously on both sides. . . Very rarely was it confined to but one side. The swelling always had the same peculiar characteristic appearance. To the eye it was smooth, but the finger easily detected three or four enlarged glands. It was elongated, about as thick as an index finger, and ran downward and forward from just below the angle of the jaw, between the body of this bone and the sterno-mastoid muscle. . . . The swelling was always tender, often painful, and frequently caused stiffness of the neck, and a choking sensation. In three-fourths of the cases there was noticed enlargement of other lymphatic glands, post-cervical, axillary, and inguinal." Another important point is the following: "All cases old enough to make complaints spoke of a choking or tight feeling in the throat, and when asked to locate the place would put the hands over both sides of and just above the larynx." Abdominal pain and tenderness were common, and in the greater number of cases the liver and spleen were somewhat enlarged. Constipation was very frequent, but in the milder cases there was some diarrhœa. The fever and other acute symptoms lasted from four to seven days, but the glands remained enlarged from nine to twenty-seven days. In none was there any rash.

Dr. Dawson Williams considers that he has seen several examples of this disease, but his confidence in the truth of Pfeiffer's description seems to have been confirmed mainly by two cases seen last March. One case, in a girl aged five years, was brought to the out-patient department of the Children's Hospital, Shadwell, for a stiff neck and swollen glands. Dr. Williams thus describes the case: "The child looked ill, had a temperature of 102.8° F., carried her head stiffly, and on the left side there was a swelling about the size of a hen's egg by which

[1] *Arch. Pediat.*, 1896, xiii. 889. [2] *Lancet*, 1897, i. 160.

the sterno-mastoid was bulged forwards. On palpation the swelling was found to consist of three glands lying beneath the muscle. They were tender, but the child appeared to suffer more if the head were moved than when the glands were manipulated gently. She had refused food, but had not complained of pain in swallowing." There was no tonsillitis, and the only noteworthy point about the throat was slight granular pharyngitis, which Dr. Dawson Williams says is extremely common in children in the East-end of London. Five days later her brother, aged three and a-half years, was seen. He presented a swelling in the neck in the same situation on the left side as that seen in his sister. There was no tonsillitis. The temperature was 102°, and he complained of some abdominal pain. Two days later he was again seen, and the glands on the right side of the neck were then also enlarged.

This paper by Dr. Williams called forth letters from Dr. Donkin[1] and Dr. Coutts,[2] both of whom had recently seen cases in which several members of a family had been attacked, and curiously enough in both instances not only children but an adult also suffered. I also sent two letters[3] referring to cases that had come under my notice. For three or four years I have been on the watch for this disease, but not until the last few weeks have any cases been seen, and it seems to me that an epidemic, at the time of writing, exists in Bristol. The first case seen was in a private patient, but fifteen others have been seen at the out-patient department of the Children's Hospital. Most of these have been brought for examination after the fever has passed off, and consequently one has had to rely more upon the history given by the mother than if one's daily routine had given the opportunity of seeing these patients earlier at their homes. The disease is, however, I think, sufficiently characteristic to be unmistakable even after the acute symptoms have disappeared. The febrile attack is depressing, and leaves the child anæmic and lethargic, and it is for listless weakness that the mother may bring the child. Without any questioning, however, she generally gives a history of symptoms of fever lasting four or five days associated with the presence of tender swellings in the neck. This tenderness may be a very marked feature, and one mother described how the child persistently kept the fingers of both hands between the neck and collar of the clothes in order apparently to steady the head and preserve the swollen glands from injury. In other cases the fever, with which there may have been delirium, has attracted more attention than the tender swellings. When, however, tenderness in the neck has been a marked feature, it has generally been noticed to have disappeared with the subsidence of the fever, and although for some time during the period of convalescence enlarged glands in the neck may be discovered they are not then as a rule tender

1 *Lancet*, 1897, i. 274. 2 *Ibid.*, 346.

3 *Ibid.*, 274, 407.

to the touch. These glands, in my experience, have been from the size of a pigeon's egg downwards, and most commonly situated beneath the anterior border of the sterno-mastoid muscle. They have been present on both sides, but have been usually larger and in greater number on one side than the other. The largest of the swollen glands has generally been just below the angle of the jaw, and below it have been two or three other glands diminishing in size from above downwards. The glands in the posterior triangles of the neck have also been frequently enlarged, but in only two cases have I found them elsewhere than in the neck, and those were in the groins. In no case have I been able to make out the enlargement of the mesenteric glands described. Next to the enlargement of the cervical lymphatic glands the most interesting feature is the difficulty of swallowing. In young children this leads to refusal of food; in older children to complaint of soreness of the throat. This difficulty of swallowing, however, is not apparently due to an actual sense of soreness. One child, two years of age, was said to have complained of having "a bone in the throat," and was further said to have "nearly choked itself" by thrusting its fingers into its throat in vain endeavours to extract the offending body, a method of procedure that does not seem to indicate great local tenderness. And children old enough to clearly express their feelings may definitely deny that there has been any pain in swallowing, and describe a sense of tightness in the throat. The most interesting observation upon this point was made by an adult, a man forty years of age, who had suffered with his eight children from fever associated with tender swellings in the neck. When describing the difficulty of swallowing he denied the existence of pain, but spoke of a sense of obstruction behind the cricoid cartilage which necessitated "a struggle to swallow." The site at which the sense of difficulty is felt is of some importance. In my experience it has been remarkable how constantly children aged four years and upwards have referred it to the level of the larynx, most commonly to the upper border of the cricoid cartilage. There has not been any lesion of the throat that would account for this abnormal sense of difficulty in swallowing. Of the other signs described, enlargement of the liver and spleen have not been definite features in the cases seen by me. In three cases the spleen was felt below the costal margin, and in a few others the liver may be said to have been enlarged, but not sufficiently to warrant the laying of any stress upon the point. While, however, splenic enlargement was not common, it is perhaps worthy of mention that in three children complaint of pain in the region of the spleen was one of the most prominent symptoms. Constipation has been a marked feature in a few cases, but the majority have been given an aperient, which has overcome any constipation that may have been present and has even produced slight diarrhœa. In two cases in which I have had opportunities for ascertaining the length of the fever, it has lasted five days, and the history in others has generally very definitely given the same duration for the acute symptoms. In one or two, however, the fever appears to have ended in four days. Besides the glandular swellings, difficulty of swallowing, and fever, a few cases have presented other symptoms. Dr. Chapman [1] in a more recent letter to the *Lancet* draws attention to the fact that five of his own children suffered from the disease, and in four there was epistaxis. He remarks that this complication does not seem to have been noticed by writers upon glandular fever. One other complication that has not been recorded, but seems to me to occur, is the occasional presence of a passing synovitis. In two cases this occurred with a relapse of the disease, and in both the synovitis was in the ankle-joint. In two others there was a history of swelling and pain in some of the finger-joints of one hand, and in another marked synovitis was present in the right sterno-clavicular joint; but in this case there had been a recent attack of acute rheumatism, and it cannot be said that this synovitis was not due to a slight intercurrent attack of that disease.

Reference has been made to the shortness of the fever; but it must not be forgotten, as Dr. Dawson Williams remarks, that the acute stage may leave behind marked anæmia and general deterioration of health, from which the child does not completely recover for a month or two. As an example it may be mentioned that in one family upon which I called, where eight children had been attacked, it was found that in one child so much anæmia and weakness remained three weeks after the disappearance of symptoms of fever, that the mother, a sensible woman, had not considered him well enough to be allowed out of doors. Dr. Park West states that so far as is known only one case in an adult has been described. Dr. Donkin and Dr. Coutts, however, in their letters to the *Lancet*, both mentioned instances in which an adult had suffered as well as children; and I also mentioned that I had met with similar cases. In one instance two adults had noticed tender swellings in the neck at the same time that three children were attacked; and in another, a father and eight children had been attacked. Indirectly I have since heard of a similar case. The mother of one of the children seen at the Children's Hospital said that the father had first suffered. Three children were afterwards attacked.

Little can be said at present about the pathology of the disease; but Dr. Dawson Williams states that it seems probable that the infective agent, whatever it may be, obtains entrance by the pharynx or tonsils without producing a local lesion. To me it appears, however, not impossible that although the pharynx may escape, the œsophagus may be in some degree

[1] *Lancet*, 1897, i. 555.

inflamed. The situation at which the difficulty of swallowing is experienced seems to suggest this. The point at which much friction must be felt in the œsophagus is just behind the cricoid cartilage. The food in passing downwards has this hard cartilage in front and the bony vertebra behind; and, as an illustration of the way these structures may affect the soft parts, it may be mentioned that it is not very uncommon in chronic debilitating diseases to find ulceration of the œsophagus at this point due apparently to pressure of this cartilage against the spinal column behind. Any inflammation of the œsophagus leading to deficient or abnormal tenacity of its mucous secretion would occasion some difficulty of swallowing, which difficulty would most likely be felt more behind the cricoid cartilage than elsewhere. And it has been to the level of this cartilage that the patients in my experience have almost constantly pointed, when asked to locate the spot at which they have felt the obstruction of which they complain.

There are other points of interest which might be considered, but my object has been little more than to direct attention to this disease, trusting that in the experience and judgment of others I may find confirmation of the truth of these few observations, cursorily given though they are.

THEODORE FISHER.

The following is a continuation of the record of **Serumtherapy** which I gave in December:

Further efforts have been made during the past year towards an effective serum-treatment of tuberculosis. Dr. Maragliano[1] has employed an anti-tuberculous serum in the treatment of pulmonary phthisis. He states that his serum contains specific antitoxins which neutralise both in animals and men the toxins of tuberculosis. It is prepared as follows: The purpose is to inject into the animals which are to furnish the serum the toxic substances that can be obtained from very virulent cultures of human tuberculosis. The first group is obtained by concentration over a sand-bath at 212° F., then by filtration through a Chamberland filter; this contains the proteins. The second, which contains the toxalbumins and a small quantity of protein, is obtained by the Chamberland filter, the culture not being heated, and concentrated in a vacuum at 86° F. Three parts of the first and one part of the second are injected into horses in proportion to the body weight, one to 500,000, and increased each day by one-half the original dose, until twenty to twenty-five times that dose is reached. The maximum dose is injected daily for six months, when immunity is reached. The treatment is now suspended for three or four weeks. The immunised animal is now bled, and the serum prepared by the

[1] *Policlin.*, 1896, iii—M., 213; abstract in *Am. J. M. Sc.* 1896, cxii. 469.

same method as for the other therapeutic serums.[1] The serum contains a specific antitoxin. One c.c. protects 1 kilogramme of guinea-pig against a lethal dose of tubercle bacilli. *In vitro* it develops bactericidal properties on cultures of tubercle bacilli.[2] The serum (in man) has slight pyrogenic properties; its effect on the temperature is the same in apyrexial tuberculous patients as in healthy men. A rise of temperature, varying in degree with the individual, regularly occurs after large doses, and is not a specific result, but the general property of the serum. It lasts two to three days, and is not accompanied by an increase of local symptoms. The rise of temperature is not proportional to the severity of the disease.

Of local symptoms, the tuberculous broncho-pneumonic patches are favourably influenced. They dry up, the moist râles disappearing: this favourable result is only to be expected when no other micro-organisms co-exist; the more extensive the association with other organisms, the less is the focus of disease influenced by the serum-treatment. Of forty-four cases with circumscribed lesions, with or without slight fever, and without extensive association with other microbes, twenty-four were dismissed as cured, the rest essentially improved by the treatment; fourteen cases with diffuse broncho-pneumonic foci, with or without fever, and with little or no affection by other microbes, were improved, some strikingly so. In diffuse broncho-pneumonias with extensive association with other microbes, and also in cases with excavation, the effect of the treatment was less favourable.

The duration of the illness is of less importance as regards successful treatment than its extent, intensity, and character. Association of the disease with diplococci and streptococci is unfavourable. Fever is also a hindrance to successful treatment; the sthenic septic fevers, resulting from a mixed infection, are very unfavourable.

The treatment is thus carried out in apyrexial cases, and in those with moderate pyrexia: an injection of 1 c.c. is given every other day for ten days, then every day for the same time; then 2 c.c. a day for ten days, and so on. In cases with high fever a dose of 10 c.c. is injected; if the fever now falls, daily injections of 1–2 c.c. are given; if it persists after eight days, a second injection of 10 c.c. is made, then a third, and so on.

Maragliano has recently published[3] an analysis of 410 cases of tuberculosis in which his treatment has been carried out by different observers. The patients were at various stages of the disease, 93 having cavities. In 98 per cent. other methods of treatment had failed. 16.2 per cent. were cured, at any rate for

[1] Abstract in *Centralbl. f. innere Med.*, 1896, xvii. 1124.

[2] *Berl. klin. Wchnschr.*, 1895, No. 32; abstract in *Centralbl. f. innere Med.*, 1896, xvii., 516.

[3] *Cron. d. clin. Med. di Genova*, 1896, iii. 2; abstract in *Progrès méd.*, 1896, 3e Sér. iii. 283.

the time, 48 per cent. were considerably improved, 25.5 per cent. remained stationary; in 8.25 per cent. of these the disease steadily progressed in spite of the treatment. He concludes that the serum treatment is not dangerous; it often cuts short the fever; it has a favourable action on the foci of disease, on the body weight, which is augmented, and it diminishes the number of bacilli; in 43 per cent. of cases they disappeared from the sputum. All the patients who had tuberculous lesions limited to the apex of one lung were cured. The serum may be employed with good prospect of success in all forms of tuberculosis.

De Renzi[1] found from a trial of the treatment in twenty-two cases no drawback to the use of the serum. In some cases a swelling of the lymph-glands in relation to the site of injection occurred; this was almost painless, and subsided without treatment, and he regards it as indicating a favourable reaction. In some cases arrest of the disease, in others improvement, occurred. Increased appetite and sense of well-being were noted. The cure is not hastened by an increase of the dose. The best results are obtained in cases of uncomplicated or limited tuberculosis without high fever.

De Renzi[2] has also investigated the action of Koch's tuberculin on patients who had undergone the serum treatment, and found that they presented a less intense reaction than those who had not been so treated, from which he concludes that they are in a certain measure rendered immune to the action of the tuberculous poison. After thirty or forty injections of serum, the reaction to doses of tuberculin as commonly employed is either absent or very slight. There was only one exception in a patient in whom the disease was very far advanced.

V. Babes and G. Proca[3] have studied the effect of a serum produced by inoculating tuberculin and dead tubercle bacilli into animals. This mode of procedure thus differs from Maragliano's, in which the poisonous products of the bacilli only are used:—

1. Dead tubercle bacilli deprived of tuberculin are passed through bodies of animals, whose serum then contains active principles which, without giving rise to fever as in the case of tuberculin, produce pathological lesions analogous to the living bacilli.

2. Tuberculin differs from this serum: (*a*) In being more toxic, the anti-tuberculous serum not being fatal even in large doses. (*b*) The serum mixed with tuberculin *in vitro* paralyses the action of the latter, except in very large doses. (*c*) Both have in common a curative action on lesions produced by dead tubercle bacilli.

[1] *Riforma med.*, 1896, xii. pt. 1, 87; abstract in *Centralbl. f. innere Med.*, 1896, xvii. 1124.

[2] *Rev. clin. e terap.*, 1896, No. 4; abstract in *Rev. de Thérap. méd.-chir.*, 1896, lxiii. 369.

[3] *Compt. rend. Acad. d. Sc.*, 1896, cxxii. 37; abstract in *Rev. de Thérap. méd.-chir.*, 1896, lxiii. 108.

3. In animals treated by tuberculin, and afterwards with dead bacilli, the serum is more potent than in those treated by tuberculin alone; the serum of animals so treated is efficacious in curing experimental tuberculosis, if injections are begun a few days after infection and several times repeated. Large doses must be used to be effectual.

4. If the poison of tuberculosis and the serum be simultaneously inoculated, no general tuberculosis follows, and the local lesion heals after a time.

5. Tuberculin can also exceptionally cure experimental tuberculosis, if the treatment is begun soon after infection. In animals treated as in 3, almost all have been saved after infection by tuberculosis. The control animals all died of tuberculosis.

6. When this anti-tuberculous serum is in contact with tubercle bacilli *in vitro*, the latter become inoffensive for guinea-pigs; in the presence of a medium favourable to growth of tubercle bacilli the medium becomes unsuitable for their growth.

So far serum-treatment in **tetanus** seems to have made no progress; cases injected after the appearance of the symptoms have died in the usual course, if they were at all severe. One observer, M. Bazy[1], whilst at the Bicêtre, observed in two or three cases tetanus follow on wounds incurred in the same locality. Since then he has employed the antitoxin serum as a prophylactic immediately on receipt of a wound.

E. Nocard[2] has endeavoured to put in practice in animals a preventive serum-treatment, seeing that injections of tetanus serum after the appearance of the first symptoms are ineffectual. Serum was distributed to veterinary surgeons with instructions in cases of animals threatened with tetanus to inject 10 c.c. of it as soon as possible after the infliction of a wound, operative or otherwise. A second dose was to be injected after fourteen days. 375 animals were thus treated; none of them suffered from tetanus, although during a period of some days to a month previously other animals in the same stable had been slaughtered on account of tetanus, and the injected animals had been left in close association with them. During the following six months none of the 375 animals died, whilst fifty-five cases of tetanus occurred in wounded animals under the same conditions which were not injected. No bad results were noticed from the injections.

Gilbert and Fournier[3] remark that the serum of animals naturally immune to **syphilis** appears to have no effect upon the course of the disease, and the same is true of the serum of tertiary syphilitics. Trial was made of the serum of two patients

[1] *Progrès méd.*, 1896, 3e Sér. iii. 138.
[2] *Bull. Acad. de Méd.*, 1895, 3e Sér. xxxiv. 407.
[3] *Med. Week*, 1895, iii. 281; see also *Internat. M. Mag.*, 1896–7, v. 260.

in the tertiary stage. 304 c.c. were injected in the course of twenty days into a patient with pronounced secondary symptoms, with the result of considerable improvement in general condition, but without disappearance of the roseolar eruption. Seventeen patients were treated with serum from animals in whom attempts had been made to produce immunity by inoculation with the blood of recent syphilitics, or with excised chancres, or with both. Seven patients received the ordinary syphilitic treatment, ten did not. Of the latter, five showed no benefit and five were more or less improved; in one there was complete disappearance of all symptoms. They conclude that the serum treatment causes improvement of general condition, disappearance of headache, and of pains in the bones and joints, and attenuation or even disappearance of the cutaneous and mucous lesions, but not constantly or invariably.

Professor Neumann injected five patients suffering from syphilis with serum from two cases in the tertiary stage of the disease. Of the five patients, two were made worse and three apparently improved; but all suffered from secondary symptoms. He tried the serum of lamb's blood in one case, without any result.

Richet[1] inoculated a woman who had had syphilis twenty years before, and was suffering from tabetic symptoms, with serum from a dog that had been inoculated eight days previously with blood from a syphilitic person. After the injections the symptoms disappeared.

Rudolph,[2] from the result of two cases, believes that erysipelas may have an extraordinarily favourable influence on syphilis. The first case was that of a man aged 52, who had acquired syphilis twenty-nine years before, and who was suffering from ulceration of the nose and supra-orbital region, which healed up nine days after the onset of erysipelas. The second case was that of a dressmaker aged 25, who had contracted syphilis at 21; had undergone several courses of mercurial treatment and taken large quantities of potassium iodide. The patient was weak and emaciated, and suffered from enlarged glands, severe pains in the knees of syphilitic origin, headache, paresis of the left side of the face, with twitching of muscles. Further treatment by potassium iodide had no effect. A severe attack of erysipelas five years after syphilitic infection resulted in cure of most of the syphilitic symptoms, so that she was able to return to work. A year after, her general health was good, but there was an enlarged gland in the submaxillary region, and a gumma on the right tibia. The author quotes Strack and Horwitz as reporting a case, and Horwitz two cases, in which recovery followed the onset of erysipelas in severe cases of syphilis in which the ordinary treatment had been of little use, and also a case of Rose's.

J. Michell Clarke.

[1] See *Med. Rec.*, 1896, xlix. 340. [2] *Centralbl. f. innere Med.*, 1896, xvii. 124.

